# Knowledge of electronic medical records system among frontline health care workers in Jos University teaching hospital, Plateau State Nigeria

**DOI:** 10.18203/2320-6012.ijrms20204867

**Published:** 2020-10-30

**Authors:** Tolulope O. Afolaranmi, Zuwaira I. Hassan, Bulus L. Dawar, Bamkat D. Wilson, Abdulbasit I. Zakari, Kayode K. Bello, Akinyemi O. D. Ofakunrin, Gabriel O. Ogbeyi

**Affiliations:** 1 Department of Community Medicine, University of Jos and Jos University Teaching Hospital, Jos Plateau State Nigeria; 2 Department of Community Medicine, Abubakar Tafawa Balewa University, Bauchi, Bauchi State Nigeria; 3 College of Health Sciences, University of Jos, Jos Plateau State Nigeria; 4 Department of Community Medicine, Jos University Teaching Hospital, Jos Plateau State Nigeria; 5 Department of Epidemiology and Community Health, College of Health Sciences, Benue State University Makurdi, Nigeria

**Keywords:** Electronic medical records, Health care professional, Knowledge, Nigeria

## Abstract

**Background:**

Electronic Medical Records system (EMRs) in any healthcare system has the potential to transform healthcare in terms of saving costs, reducing medical errors, improving service quality, increasing patients’ safety, decision-making, saving time, data confidentiality, and sharing medical. Evidence on the current state of EMR system in Nigeria health system particularly its knowledge among health professionals is limited. Hence, this study was conducted to assess the level of knowledge EMRs among frontline health care workers in a tertiary health institution in Jos, Plateau State.

**Methods:**

This was a cross-sectional study conducted between April and August 2019 among 228 frontline health care workers in Jos University Teaching Hospital using quantitative method of data collection. SPSS version 20 was used for data analysis and a p-value of ≤ 0.05 considered statistically significant.

**Results:**

The mean age of the respondents in this study was 35±8 years with 93 (40.8%) being 36 years and above. The overall level of knowledge of EMRs was adjudged to be good among 163 (71.5%) of the participants. Category (pharmacists) of the respondents was found to influence good knowledge of EMRs (OR=1.37; 95% CI=1.007–1.865; p=0.045).

**Conclusions:**

This study has demonstrated a relatively high level of good knowledge of EMRs with variation existing along the categories of health care workers bringing to light the existence of a good knowledge base in the light of future EMRs implementation.

## INTRODUCTION

Electronic medical records (EMR) refers to the electronic record of health-related information of individuals created, complied, managed and used by authorized health care providers in a health institution. ^1–3^ Studies have shown that the adoption of an EMR system in any healthcare system has the potential to transform healthcare in terms of saving costs, reducing medical errors, improving service quality, increasing patients’ safety, decision-making, saving time, data confidentiality, and sharing medical information.^4–12^ EMR systems in most developing countries such as Nigeria is in its early stage due to inadequate healthcare infrastructures, health professionals’ attitude and awareness level, lack of proper management, resource shortage, skill related issues, users’ resistance, policy related issues, poor commitments of staffs, and poor maintenance services among others.^6,13^

Timely and accurate access to patient’s information which EMRs provides are essential in meeting the healthcare needs of any patient culminating into sound clinical decisions and if otherwise the resultant effect could be inefficient patient care.^14,15^ The critical need for good health information systems in sub-Saharan Africa has become the current focus of attention as the region accommodates about 12% of the world’s population and accounting for 27% of the global burden of disease.^8–10^ Yet, this region still lags behind in Health Information Technology (HIT) which is vital in ensuring improved patients care despite its growing interest globally.^3,8–10^ Evidence on the current state of EMR system (EMRs) in Nigeria health system particularly its knowledge among health professionals who are the fulcrum of its implementation is limited making it imperative to conduct a study to assess the level of knowledge EMRs among frontline health care workers in a tertiary health institution in Jos, Plateau State.

## METHODS

### Study setting

This study was conducted in Jos University Teaching Hospital (JUTH), a tertiary health institution founded in 1975 and affiliated with the University of Jos.^16,17^ It is located in the Lamingo Area of Jos North Local Government Area (LGA) with an estimated bed capacity of about 600.^17^ JUTH offers a vast variety of specialized services in the various aspects of healthcare, research and training and serves as a referral center to the surrounding states in the North central, part of North western and eastern part of Nigeria.^17^ JUTH being a tertiary health facility has following service delivery units; surgery, internal medicine, obstetrics and gynaecology, paediatrics, community medicine, radiology, ophthalmology, pathology, laboratory medicine, otorhinolaryngology, anaesthesia, psychiatry and dentistry among others.

### Study population

The study population comprised of all frontline health workers across all categories in all clinical departments including the Accidents and Emergency (A&E) units of Jos University Teaching Hospital. The frontline health care workers in the study included, resident doctors, medical officers, pharmacists, nurses and midwives.

### Study design

A cross-sectional study design conducted between April and August 2019 to determine the knowledge of EMRs among the frontline health care workers in Jos University Teaching Hospital, Plateau State Nigeria using quantitative method of data collection

### Sample size estimation

The sample size for this study was determined using the appropriate sample size determination formula for a cross sectional study.^18^ Where n is the minimum sample size, Z is the standard normal deviate at 95% confidence interval (1.96), q is the complementary probability (1-p), d is the precision of the study set at 0.05 and p is the proportion (82.9%) of respondents with good knowledge from previous similar study.^19^ This gave a sample size of 228 after addition of 5% to cater for non, poor and or incomplete responses.

### Criteria for inclusion in the study

All resident doctors, medical officers, pharmacists, nurses and or midwives full time employees of the hospital, present at the time of the study and had spent at least one year on the jobs who had given consent for participation were included in the study. One (1) year period was set as cut-off for inclusion as it would ensure that the participants would have had sufficient interaction with the medical record keeping system in the institution.

### Sampling technique

A stratified sampling technique was used in view of the fact that the categories of frontline health care workers had differing number of participants. Lists of all the categories (resident doctors, medical officers, pharmacists, nurses and midwives) of health care workers in the hospital with their institutional identification numbers and the designated units/department of primary assignments were obtained from the designated coordinating units forming the sampling frame. Following which proportion to size technique was used to obtain the number of participants to be sampled from each of the categories. This was done by dividing the number of participants who had met the inclusion criteria (resident doctors - 330, medical officers - 100, nurse and midwives-536 and pharmacists - 37) by the cumulative total number of all frontline health care workers who had met the inclusion criteria (1003) multiplied by the sample size of 228. This gave the following number health care workers to be sampled per category: resident doctors - 75, medical officers - 23, nurse/midwives-122 and pharmacists-8). Thereafter, the serialized lists of the categories of health care workers was used as the sampling frames respectively from which computer generated table of random numbers using WINPEPI statistical software was used to select estimated number of participants per category respectively without replacement. These participants were then sampled in their respective units/departments of primary assignment for a period of two months. For those who declined consent for participation, repeat selections using the table of random numbers were done until the sample size was met.

### Data collection instrument

A semi-structured self administer questionnaires adapted from previous studies comprising of three sections; socio-demographic characteristics, knowledge of EMRs and factors influencing EMRs knowledge workplace handling practices was used in obtaining information from the study participants.^20^ Three research assistants were trained on the content and method of administration of questionnaire prior to the commencement of the study by the leader researcher and his team. The data collection instrument was pretested in among health care workers in Plateau state specialist hospital Jos among 10% of the calculated minimum sample size. This helped to address ambiguity in the questions, estimate administration time for proper planning for data collection and assess appropriateness of the contents in addressing the objectives of the study.

### Data collection procedure

The participants were given the questionnaire in their respective units/departments and subsequently retrieved an hour later for most of them and at the close of work for those who were not able to complete the questionnaire within the hour by the trained research assistants. Following the retrieval of the filled data collection instrument form the participants, the trained research assistant reviewed all the questionnaires for completeness and appropriateness of the responses as required. The questionnaires not completely filled were returned for proper filling and retrieved back. This short duration of time (1 hour) was used as an internal control mechanism to ensure that all questionnaires were retrieved and to also minimize the possibility information sharing among the respondents.

### Grading of response

Definition of EMR was adjudged correct if the respondents provided information encompassing the following: electronic record of health-related information of individuals created, complied, managed and used by authorized health care providers in a health institution.^1,2^

A total of 6 questions were used to assess the level of knowledge with 2 marks allotted for every correct response and one for incorrect response giving a total attainable score of 12. A percentile graph of the score was plotted and scores less than the 50^th^ percentile was considered as poor knowledge while scores 50^th^ percentile and above was considered as good knowledge.

### Data analysis

The data obtained were processed and analyzed using SPSS version 20 where socio-demographic characteristics of the respondents such as age group, sex, marital status etc were expressed in frequency and percentage. Mean±standard deviation were used as summary indices for age and the knowledge scores of the respondents while median and interquartile range for summarizing the duration of practice after demonstration of skewness.

The outcome variable in the study was the level of knowledge of EMRs categorized as good and poor and presented in frequency and percentage. Binary logistic regression was used to identify predictors of good knowledge of EMRs where crude odds ratio and 95% confidence interval were used as point and interval estimates of the effects of the independent variables on good knowledge of EMRs in the logistic regression model. A probability value of less than 0.05 was considered statistically significant in this study.

## RESULTS

The mean age of the respondents in this study was 35±8 years with 93 (40.8%) being 36 years and above. Assessment of the sex distribution showed that females accounted for 107 (46.9%) of the respondents while the corresponding 121 (53.1%) were males. The median duration of practice as health care professionals was 3 (IQR: 2–6) years as most (74.1%) of them reported to have been in practice for 5 years or less. With regards to attendance of any form of training on EMRs, only 35 (15.4%) had ever attended any EMRs related trainings (Table 1).

In this study, 189 (82.9%) of the study participants were aware of EMRs while 167 (73.2%) of them could correctly define electronic medical records. With regards to the areas application of EMRs, 211 (92.5%) of the respondents mentioned patients’ medical records, while archiving of laboratory results, treatment management as well as data management and repository were mentioned by 189 (81.6%), 160 (70.2%) and 106 (46.5%) respectively. Furthermore, reduction in occurrence of medical errors, improvement in confidentiality of patient care, improvement in quality of care and reduction in cost of health care were expressed by 93.9%, 92.1%, 90.8% and 21.5% of the participants respectively as some of the advantages of the use of EMRs. Additionally, the overall level of knowledge of EMRs was adjudged to be good among 163 (71.5%) of the participants with a mean knowledge score of 9.8±2.6 out of 12 (Table 2).

Assessment of factors influencing the level of knowledge of EMRs among the respondents in the study revealed that the knowledge of EMRs varied along the category of the health care workers as the odds of good knowledge of EMRs among the pharmacists was 1.37 times the odds among the resident doctors which was significant statistically (95% confidence interval: 1.007–1.865; p=0.045).

However, the odds of good knowledge of EMRs among medical officers was 0.50 times (95% confidence interval: 0.196–1.261; p=0.141) the odds among the resident doctors while of the nurse/midwives was 0.67 times (95% confidence interval: 0.324–1.378; p=0.275) the odds of good knowledge of EMRs among the resident doctors. Furthermore, the odds of good knowledge of EMRs among those who had ever attended EMRs related trainings was 1.18 times the odds of those who had not attended any such trainings (95% confidence interval:0.541–2.573; p=0.678). Other factors such as age, duration of practice and sex were found not to have any statistically significant influence of the knowledge of EMRs (Table 3).

## DISCUSSION

Electronic medical records system is one of the essential pathways to achieving and optimizing the delivery of quality of health care and a veritable repository platform for clinical data for biomedical research.^3,6^

However, its implementation is at differing levels globally with most countries in sub-Saharan Africa yet to implement while those who have implemented are still at the early phase. Importantly, this study setting has not implemented EMRs and it is imperative to assess the knowledge EMRs among its frontline health care workers as its utilization upon implementation in the nearest future would be hinged on the knowledge base of this workforce.

Understanding of the concept of EMRs is fundamental to gaining insight into participants’ knowledge of EMRs. In this study, majority of the participants could correctly define EMR which is in synergy with the findings of studies conducted in Nigeria and Kuwait.^19,21^ This finding could be attributable to the fact that this study was conducted in a tertiary health institution where continuous medical education is a pre-requisite for constancy of engagement and renewal of annual practising licenses which may have endeared them to acquiring information on EMRs.

Furthermore, the similarity this study shares with other cited studies further corroborates the fact health care workers are making deliberate and self-driven efforts to keep in tune with the global shift from conventional paper based medical records systems to the electronic health information systems particularly in settings where it not in use.

Knowledge of EMRs and other related electronic health information systems could be essential to optimum utilization of such systems when put in place. In this study, more than two-thirds of the participants demonstrated good knowledge of EMRs though in the absence of a functional electronic medical records system. Studies conducted in Ethiopia, Kuwait and Nigeria reported similar findings though slight variations existed in the methodology of assessment used.^19,20,22,23^

Contrary to the finding of this study, lower levels of knowledge of electronic medical records and information systems were reported in studies conducted in South Africa, Nigeria and Saudi Arabia.^24–26^ The finding of this study could also be a reflection of the level of access to information and its utilization in this era of unlimited access to medical information. The implication of the findings of this study to practice is that a relatively good knowledge base of EMRs exits which will provide a good and smooth take off of such system when it is eventually implemented.

Additionally, in view of the reported level of knowledge of EMRs in this study, it will be important for other studies be conducted to assess the level of readiness of the health care to using EMRs so as to provide a holistic EMRs pre-implementation assessment. Furthermore, variation exists in the level of knowledge of EMRs along the categories of health care workers in this study as the pharmacists demonstrated a higher likelihood of good knowledge of EMRs.

This brings to light, the need to take into consideration peculiarities of the categories of health care workers at implementation of EMRs particularly with regards to relevant trainings and information sharing. It is imperative to state that a self assessment approach was employed through self administration of questionnaires and it is a commonplace that respondents would use all means possible to provide the most favourable response which may have impacted on the of knowledge EMRs reported.

## CONCLUSION

This study has demonstrated a relatively high level of good knowledge of EMRs with variation existing along the categories of health care workers bringing to light the existence of a good knowledge base of EMRs in the light of its future implementation in this setting.

## Figures and Tables

**Table 1:** Socio-demographic characteristics of the respondents.

Characteristics	Frequency	Percentage
**Age group (in years)**		
≤35	135	59.2
36 and above	93	40.8
Total	228	100.0
	Mean±SD	
**Mean age**	35.0±8.0 years	
**Sex**		
Female	107	46.9
Male	121	53.1
Total	228	100.0
**Marital status**		
Single	67	29.4
Married	161	70.6
Total	228	100.0
**Duration of practice (years)**		
≤ 5	169	74.1
6 and above	59	25.9
Total	228	100.0
**Median duration of practice**	Median (IQR) 3 (2–6) years	
**Category of health care workers**		
Medical officers	23	10.1
Residents Doctors	75	32.9
Pharmacists	8	3.5
Nurse/midwives	122	53.5
Total	228	100.0
**Attendance of training on EMRs**		
Ever attended	35	15.4
Never attended	193	84.6
Total	228	100.0

SD=Standard Deviation, IQR=Inter-quartile Range

**Table 2:** Knowledge of the respondents on EMRs.

Characteristics	Frequency	Percentage
**Awareness of EMRs**		
Yes	189	82.9
No	39	17.1
Total	288	100.0
**Definition of EMRs**		
Correct	167	73.2
Incorrect	61	26.8
Total	288	100.0
Areas of application of EMRs*		
Patients’ record	211	92.5
Laboratory results	189	81.6
Treatment/drug management	160	70.2
Data management and repository	106	46.5
Advantages of EMRs*		
Reduction in workload	98	43.0
Improvement in confidentiality of care	210	92.1
Reduction in medical errors	214	93.9
Improvement in quality of health care	207	90.8
Reduction in health care cost	49	21.5
Reduction in waiting time	178	78.1
**Knowledge of EMRs**		
Good	163	71.5
Poor	65	28.5
Total	228	100.0
	Mean±SD	
**Knowledge scores**	9.8±2.6 out of 12	

* Multiple responses elicited, SD=Standard Deviation

**Table 3:** Logistic regression of predictors of good knowledge of EMRs.

Factors	COR	95% Confidence Interval	P value
**Age group (years)**			
≤35	0.96	0.532 – 1.717	0.878
>36 and above	1	-	-
**Sex**			
Female	0.81	0.453–1.434	0.464
Male	1	-	-
**Duration of practice (years)**			
≤ 5	0.49	0.235–1.013	0.054
6 and above	1	-	-
**Marital status**			
Single	1.34	0.723–2.489	0.351
Married	1	-	-
**Attendance at EMRs training**			
Ever attended	1.18	0.541–2.573	0.678
Never attended	1	-	-
**Category of health care workers**			
Medical officers	0.50	0.196–1.261	0.141
Nurse/midwives	0.67	0.324–1.378	0.275
Pharmacists	1.37	1.007–1.865	0.045
Resident doctors	1	-	-

COR=Crude Odds Ratio
